# LC–MS Based Metabolomics Study of the Effects of EGCG on A549 Cells

**DOI:** 10.3389/fphar.2021.732716

**Published:** 2021-09-28

**Authors:** Tingyu Pan, Di Han, Yong Xu, Wenpan Peng, Le Bai, Xianmei Zhou, Hailang He

**Affiliations:** ^1^ Affiliated Hospital of Nanjing University of Chinese Medicine, Nanjing, China; ^2^ Department of Respiratory Medicine, Jiangsu Province Hospital of Chinese Medicine, Nanjing, China; ^3^ Arizona Metabolomics Laboratory, College of Health Solutions, Arizona State University, Scottsdale, AZ, United States

**Keywords:** EGCG, A549 cells, metabolomics, arginine and proline metabolism, glutamate metabolism, histidine metabolism

## Abstract

(−)-Epigallocatechin-3-gallate (EGCG) is the main bioactive catechin in green tea. The antitumor activity of EGCG has been confirmed in various types of cancer, including lung cancer. However, the precise underlying mechanisms are still largely unclear. In the present study, we investigated the metabolite changes in A549 cells induced by EGCG *in vitro* utilizing liquid chromatography-mass spectrometry (LC-MS)-based metabolomics. The result revealed 33 differentially expressed metabolites between untreated and 80 μM EGCG-treated A549 cells. The altered metabolites were involved in the metabolism of glucose, amino acid, nucleotide, glutathione, and vitamin. Two markedly altered pathways, including glycine, serine and threonine metabolism and alanine, aspartate and glutamate metabolism, were identified by MetaboAnalyst 5.0 metabolic pathway analysis. These results may provide potential clues for the intramolecular mechanisms of EGCG’s effect on A549 cells. Our study may contribute to future molecular mechanistic studies of EGCG and the therapeutic application of EGCG in cancer management.

## Introduction

As one of the most common cancers globally, lung cancer causes a severe social burden (Ferlay et al., 2015; Siegel et al., 2016). Worldwide, an estimated 2.2 million new lung cancer cases occurred in 2020 (Sung et al., 2021). As the second most commonly diagnosed cancer, lung cancer remained the leading cause of cancer death, with an estimated 1.8 million deaths in 2020 (Sung et al., 2021). Despite significant advances that have been made in the interventions, including surgery, radiation therapy, chemotherapy, targeted therapy, and immunotherapy on lung cancer, the 5 years survival of lung cancer only remains 21% (Miller et al., 2019; Siegel et al., 2021). It is critical to consider other preventive and therapeutic measures for lung cancer not only to decrease its incidence and mortality but also to overcome the toxicity, side effects, and cost of existing treatments (Hirsch et al., 2017).

Epigallocatechin-3-gallate (EGCG) is the most abundant and effective catechin in numerous types of white tea and green tea (Sano et al., 2001; Tang et al., 2019). It has been shown that EGCG can inhibit tumor growth and stimulate cancer cell apoptosis in various human cancers *in vivo* and *vitro* studies (Huh et al., 2004; Chen et al., 2016; Yang et al., 2016; Huang et al., 2017; Liu et al., 2017; Borutinskaite et al., 2018; Gan et al., 2018; Zhou et al., 2018; Wei et al., 2019; Wu et al., 2019; Yoshimura et al., 2019; Almatroodi et al., 2020; Panji et al., 2021; Romano and Martel, 2021). Many studies have demonstrated that EGCG may take a role in the initiation, promotion, and progression of cancer through the modulation of various mechanisms, including cellular proliferation, differentiation, apoptosis, angiogenesis, and metastasis, which leads to its anticarcinogenic activities (Chen and Dou, 2008; Ma et al., 2013; Luo et al., 2014; Luo et al., 2017; Moradzadeh et al., 2018; Pal et al., 2018; Li et al., 2019; Almatroodi et al., 2020; Yin et al., 2021). Increasing evidence has shown that EGCG possesses anti-tumorigenic property against non-small cell lung cancer (Sonoda et al., 2014; Shi et al., 2015; Gu et al., 2018; Hu et al., 2019). Even though the antitumor activity of EGCG in lung cancer has been extensively investigated, the underlying mechanism remains unclear.

Metabolomics is an exciting tool to detect small metabolic compounds and monitor small global molecule endogenous metabolite changes induced by biochemical reactions in biological systems (Dunn et al., 2011; Griffin et al., 2011; Reaves and Rabinowitz, 2011; Gu et al., 2012; Carroll et al., 2015; Zampieri et al., 2017; McCartney et al., 2018; Yan and Xu, 2018; Yeung, 2018; Shi et al., 2019; Eghlimi et al., 2020; He et al., 2020; Lim et al., 2020; Wei et al., 2021). Metabolomics is a promising approach to searching potential biomarkers and novel therapeutic strategies for lung cancer (Luengo et al., 2017; Seijo et al., 2019; Noreldeen et al., 2020; Schmidt et al., 2021). To date, the precise molecular mechanisms of antitumor activity in lung cancer induced by EGCG still keep unclear and need more investigation, especially from a metabolism point of view. Therefore, in this study, we applied liquid chromatography-mass spectrometry (LC-MS) based metabolomics and employed A549 cells as an *in vitro* model to further explore the effect of EGCG on lung cancer cell metabolism.

## Materials and Methods

### Reagents and Materials

EGCG was purchased from Sigma-Aldrich (St. Louis, MO, United States). A549 cell line (ATCC NO. CCL-185) was purchased from American Type Culture Collection (ATCC, Manassas, VA, United States). The Cell Counting Kit-8 (CK04-05) was obtained from Dojindo Molecular Technologies (Gaithersburg, MD, United States). 4′,6-diamindino-2-phenylinodole (DAPI) staining kit was purchased from FcmacsBiotechCo., Ltd. (Jiangsu, China). BCA™ Protein Assay Kit was obtained from Thermo Scientific (Waltham, MA, 84 United States). LC-MS-grade isopropanol (IPA), acetonitrile (ACN), MeOH, and CH_2_Cl_2_ were purchased from Fisher Scientific (Pittsburgh, PA). HPLC grade acetic acid, U-^13^C glucose, N-tert-butyldimethylsilyl-N-methyltrifluoroacetamide (MTBSTFA), methoxyamine hydrochloride, ammonia acetate, anhydrous pyridine, and dimethyl sulfoxide (DMSO) and all of the standard compounds used for metabolic identification were acquired from Sigma-Aldrich (St. Louis, MO, United States).

### Cell Culture

The A549 cells were cultured in Dulbecco’s modified eagle medium (DMEM) (Corning, 10-013-CV) supplemented with 10% fetal bovine serum (FBS) (Corning, 35-010-CV). Cells were grown at 37°C and 5% CO_2_ in a humidified atmosphere.

### Cell Viability Assay

Cells were seeded in 96-well plates (8.0 × 10^3^ per well) and treated with different concentrations of EGCG (20, 40, 60, 80, 100, 120, 160, 200 μM) for 24 h. After treatment, the viability of A549 cells was measured via the Cell Counting Kit-8 assay. Briefly, 10 μl of CCK-8 reagents were inserted into each well before incubation in an incubator with 5% CO_2_ at 37°C for 3 h. Subsequently, absorbance at 450 nm was measured using a microplate reader (Molecular Devices, CA, United States). Viability is expressed as a cell activity percentage between the EGCG group and the control group.

### LC-MS Metabolomics Analysis

In this study, we utilized a pathway-specific LC-MS method that can cover more than 300 metabolites from >35 metabolic pathways (Carroll et al., 2015; Gu et al., 2015; Sperber et al., 2015; Li et al., 2018; Jasbi et al., 2019; Liu et al., 2019; Shi et al., 2019). Briefly, A549 cells were seeded in 6-well plates (4.5× 10^5^ cells/well) with 10% FBS supplemented with DMEM. Then, cells were incubated overnight in an incubator with 5% CO_2_ at 37°C. The cells were then treated with EGCG for 24 h. For sample preparation, the cells were first rinsed with PBS. Then 1.2 ml of 80% MeOH was added into each well for extraction of intracellular metabolites. Samples were completely lysed using an ultrasonic homogenizer in an ice bath for 20 min and centrifuged at 14,000 rpm under 4°C for 10 min. Following that, 500 μl of each supernatant was retained and dried under vacuum for 4 h. The dried samples were reconstituted using 150 μl of solvent (PBS: ACN = 4:6) and then centrifuged at 14,000 rpm under 4°C for 10 min. Sets of samples of identical volume were combined for quality-control specimens solvent (B) to assess instrument performance.

The supernatants were analyzed by liquid chromatography-mass spectrometry (LC-MS) simultaneously after centrifugation. 100 μl of the supernatant was transferred to a new vial and analyzed by an Agilent 1290 LC-6490 Triple Quadrupole mass spectrometer system equipped with an electrospray ionization (ESI) source. LC was performed on a Waters XBridge BEH Amide column (150 × 2.1 mm, 2.5 µm particle size, Waters Corporation, Milford, MA). The mobile phase for chromatographic separation was composed of solvent (A): 10 mM ammonium hydroxide, 10 mM ammonium acetate in 95% H_2_O/5% ACN, and solvent (B): 10 mM ammonium hydroxide, 10 mM ammonium acetate in 95% ACN/5% H_2_O. Following a 1 min isocratic elution of 90% solvent (B), solvent (B) was gradually reduced to 40% in 10 min (*t* = 11 min) and then kept at 40% for 4 min (*t* = 15 min). Subsequently, solvent (B) was returned to 90% to run the next sample. Each sample was injected twice, 4 μl for positive ion electrospray ionization analysis and 10 μl for negative ion analysis. Multiple reaction monitoring (MRM) mode was employed for targeted data acquisition.

The QQQ-MS system was operated with a capillary voltage of 3.5 kV. The nebulizer gas (N2) pressure was set at 30 psi with a drying gas (N2) flow rate of 15 L/min, and the temperature was 175°C. The flow rate of sheath gas (N2) was set to 11 L/min with a temperature of 225°C. A CE range of 5–50 V in increments of 5 V, and 4 CAV values (2 V, 4 V, 6 V, 8 V) were evaluated for MRM optimization; optimized CE and CAV values were determined from the highest MRM response.

The software programs used to control the LC-MS system and integrate extracted MRM peaks were Agilent MassHunter Workstation and Agilent MassHunter Quantitative Data Analysis, respectively. Protein concentrations in each sample were utilized to normalize metabolite levels.

The post-preparative stability of the sample was tested by running five prepared quality control (QC) samples kept in an autosampler (maintained at 4°C). In addition, one QC sample was inserted every 3–4 test samples during the whole process to validate system suitability and stability.

### Statistical Analysis

Measurement data are the mean ± standard deviation (SD) and analyzed via the Student’s two-tailed *t*-test or one-way analysis of variance (ANOVA) with Tukey’s post hoc analysis, and *p* < 0.05 was considered as a significant difference.

Principal component analysis (PCA), partial least squares discriminant analysis (PLS-DA), pathway analysis overview, and heatmap clustering of altered metabolic profiling analysis were performed using MetaboAnalyst 5.0 (https://www.metaboanalyst.ca/). In pathway analysis, “*Homo sapiens* (KEGG)” library was selected, as well as Hypergeometric test for pathway enrichment analysis and relative betweenness centrality for pathway topology analysis.

## Results

### EGCG Suppressors Cell Viability of A549 Cells

**FIGURE 1:**
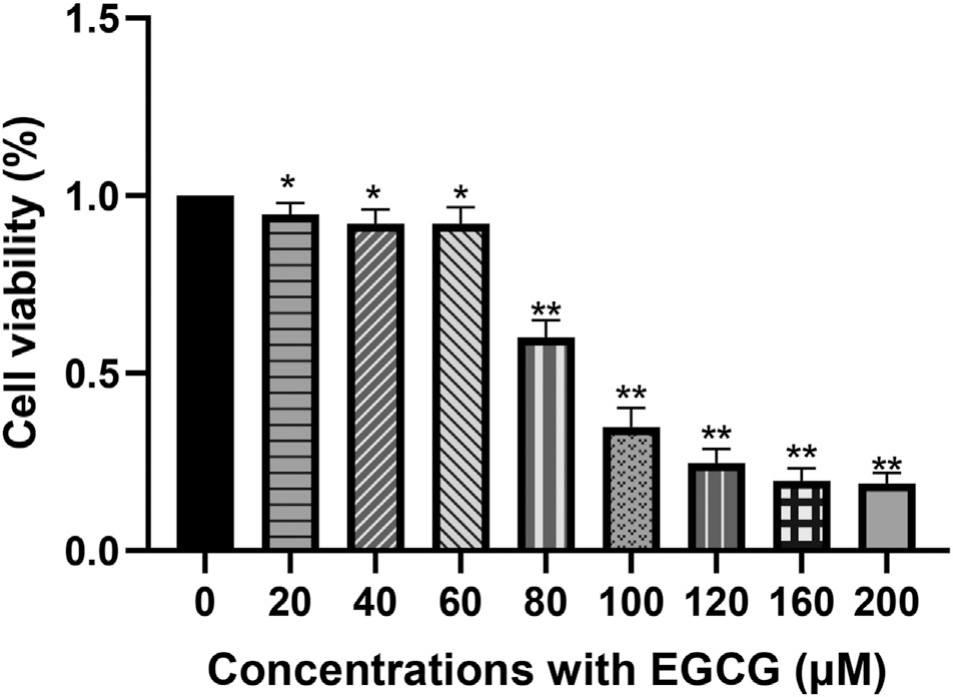
Cell viability of A549 cells after EGCG exposure. A549 cells were exposed to 20–200 μM EGCG for 24 h, and cell viability was determined utilizing a CCK-8 assay. The experimental data are expressed as the mean value with SD of three independent replicates. (*p < 0.05, **p < 0.005).

After exposure to EGCG for 24 h, the A549 cell viability was downregulated in a dose-dependent manner within the concentration range of 60–100 μM (Figure 1). Cell viability was 92.10 ± 3.23% at 40 μM (*p* < 0.05) and reduced to 60.01 ± 4.02% at 80 μM (*p* < 0.005). As shown in Supplementary Figure S1, abnormal nucleus margin and shortening of nucleus diameter occurred at EGCG exposure groups, especially at the concentrations of 100 μM. Considering the balance between cell viability and data interpretability, a concentration of 40 μM was chosen for further experiments. The concentration of 80 μM was also selected in the subsequent metabolomics experiments to help capture more responses on cell metabolism related to the effect of EGCG.

### LC-MS Metabolite Profiling of A549 Cells After EGCG Treated

#### LC-MS of Metabolic Profiles

**FIGURE 2:**
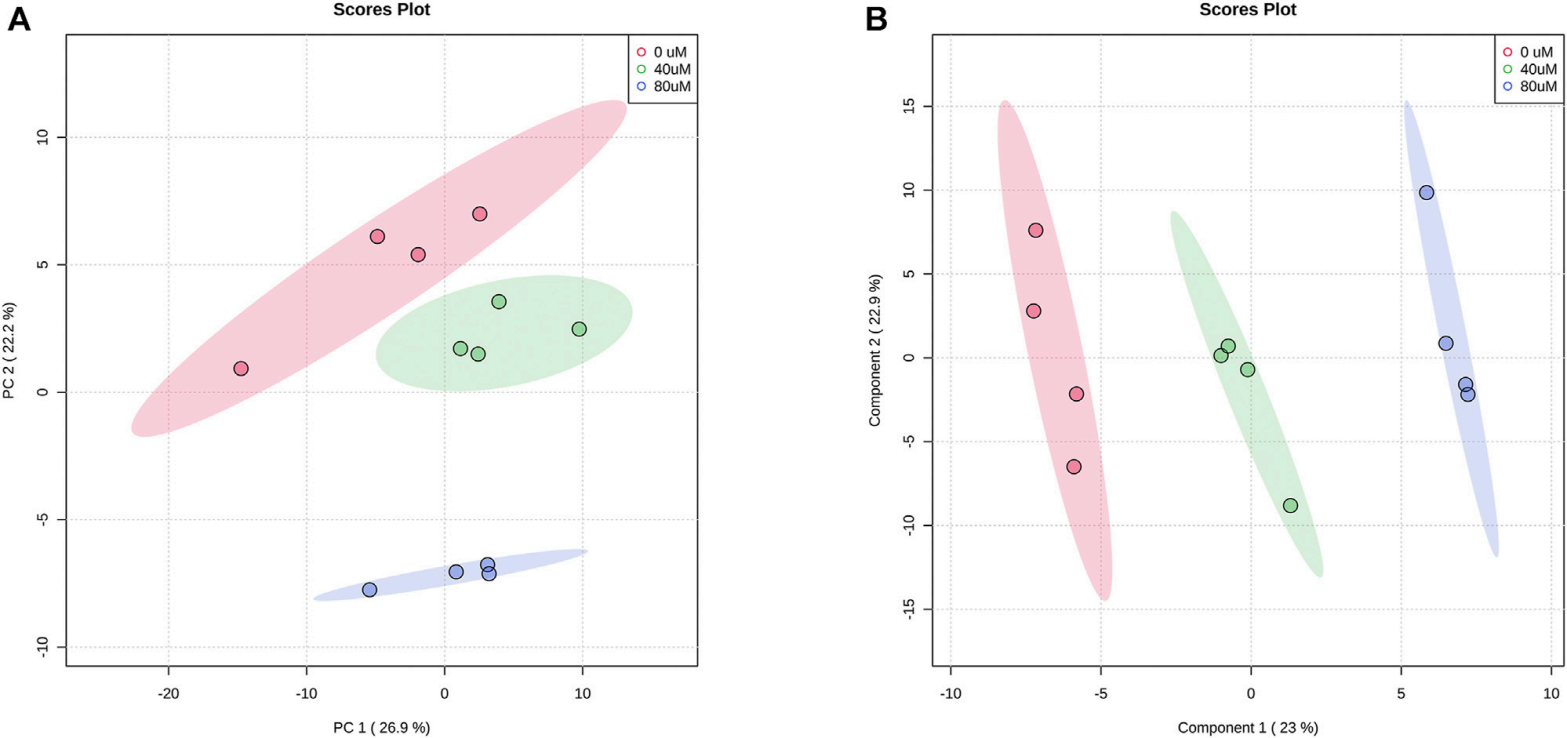
Score plots of PCA **(A)** and PLS-DA **(B)** models for the metabolome data obtained by LC-MS, showing the metabolic profile differences between control and EGCG-treated groups. Red cycle: control group; green cycle: 40 μM EGCG-treated group; blue cycle: 80 μM EGCG-treated group.

In total, we found that 173 metabolites were reliably detected with relative abundances >1,000 in more than 80% of all samples. After normalization by averaged values from the QC injection data, 142 metabolites had a coefficient of variation (CV) value of <30%. We analyzed the metabolic profiles of these 142 metabolites of 40 μM, 80 μM EGCG-treated and untreated A549 cells. Based on the LC-MS data, the PCA score plot of metabolites showed an obvious separation among the control group, 40 μM EGCG-treated group, and 80 μM EGCG-treated group (Figure 2A). No outlier detection was performed from the data overview. PLS-DA was further undertaken to reveal the metabolic deviations between the EGCG-treated groups and the control group. As shown in Figure 2B, the metabolite profiles of the three groups were distributed in significantly separated clusters. Although it is a supervised classification method, component 1 and component 2 in the PLS-DA model (Figure 2B) accounted for 23 and 22.9% of the total variance in the data respectively, which indicated that significant metabolic disturbances were induced in A549 cells treated by EGCG.

**FIGURE 3:**
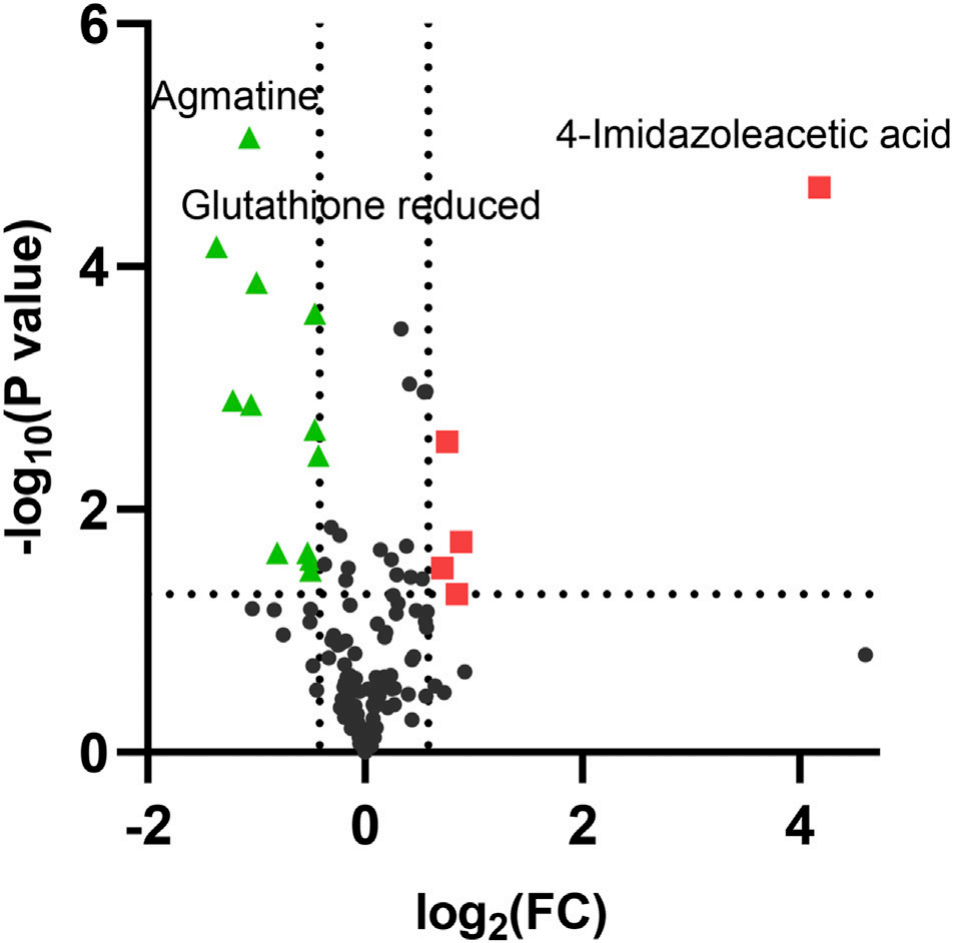
Volcano plot analysis of differential metabolites in A549 cells after exposure to 80 μM EGCG. The *x*-axis represents log_2_ (fold change), while the *y*-axis represents *p*-value in -log_10_ scale. The significantly up-regulated metabolites were indicated in red squares and down-regulated in green triangles. (*p* < 0.05 and fold change >1.5 or <0.75).

**FIGURE 4:**
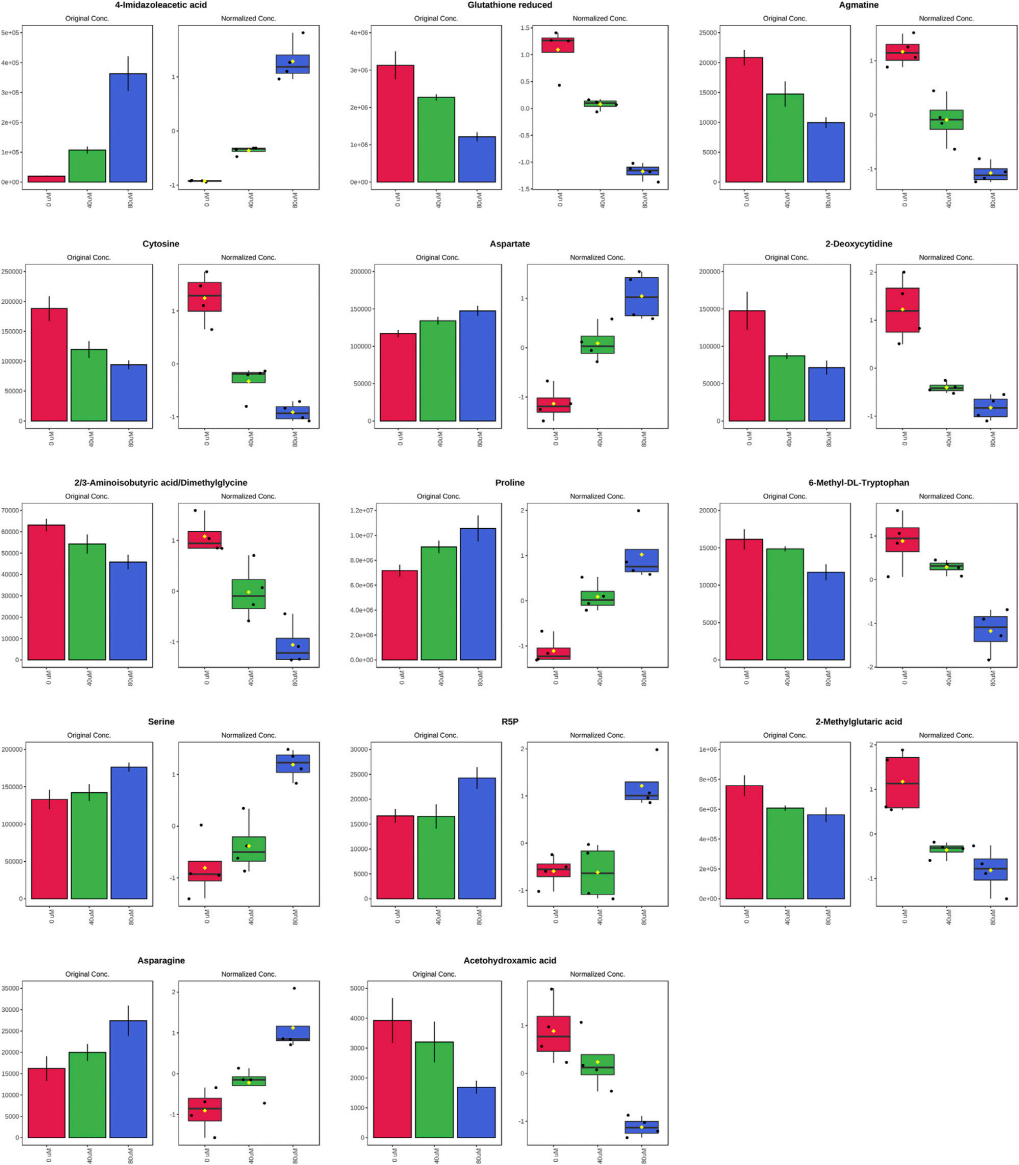
Significantly altered metabolites among the control and the EGCG-treated groups. The bar plots on the left show the original peak intensity values (mean ± SD). The box and whisker plots on the right summarize the normalized values.

**FIGURE 5:**
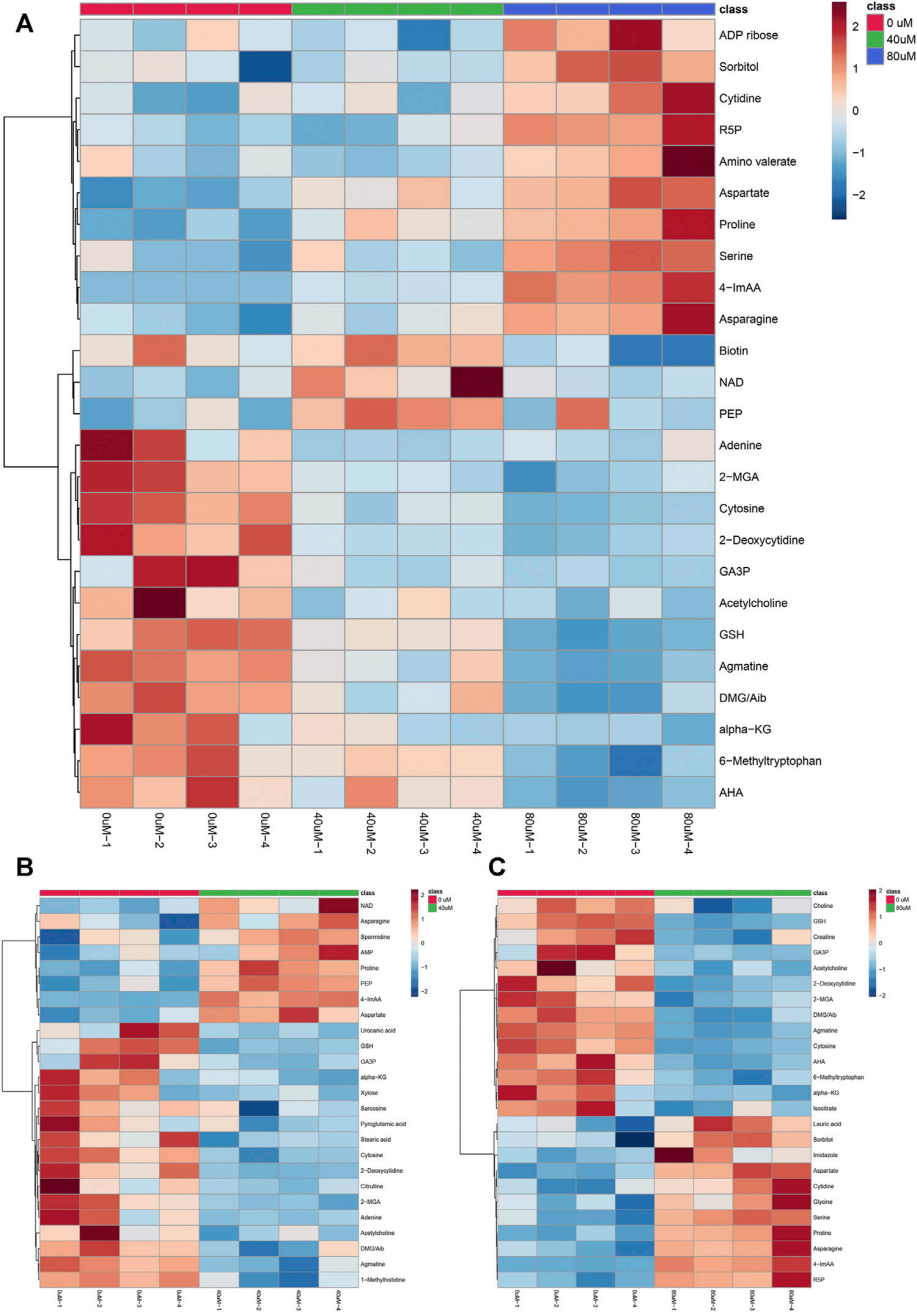
Hierarchical clustering heat map of the top 25 differential metabolites selected based on t-tests/ANOVA, with the degree of change marked with red (up-regulation) and blue (down-regulation). The distance measure was set to “Euclidean” and the clustering algorithm was set to “Ward”. **(A)**: control group, 40 μM EGCG-treated group and 80 μM EGCG-treated group; **(B)**: control group and 40 μM EGCG-treated group; **(C)**: control group and 80 μM EGCG-treated group. **Abbreviations:** R5P, Ribose-5-phosphate; 4-ImAA, 4-Imidazoleacetic acid; NAD, nicotinamide adenine dinucleotide; PEP, phosphoenolpyruvate; 2-MGA, 2-Methylglutaric Acid; GA3P, glyceraldehyde 3-phosphate; GSH, glutathione; DMG, Dimethylglycine; Aib, 2/3-Aminoisobutyric acid; alpha-KG, alpha-Ketoglutaric acid; AHA, Acetohydroxamic acid.

As the volcano plot shows (Figure 3, Supplementary Figure S2), the up-regulated metabolites between the control and the EGCG exposure groups were presented on the right-hand side of the valley, while the left-hand side of the valley represents those that were down-regulated. The number of significantly altered metabolic abundances in the 80 μM group was greater than that in the 40 μM group, which indicated that EGCG disturbed the A549cells in a dose-dependent manner. The ANOVA test analysis was utilized to identify potential biomarkers contributing most to the difference between control and the EGCG-treated groups. The results with metabolites (*p* < 0.05) are shown in Table 1 and separately in Figure 4. The top 25 significantly changed metabolites were visualized using a heat map in a red-blue scale (from higher to lower metabolite levels) (Figure 5). In pairwise comparison, metabolites with a *p*-value below 0.05 and fold change above 1.5 or below 0.75 were selected as potential biomarkers. As shown in Supplementary Table S1, the identified metabolites were summarized, and a total of 11 features were selected as potentially altered metabolite markers in A549 cells exposed to 80 μM EGCG compared with the control group. In addition, all of the disturbed metabolites with *p* < 0.05 in 80 μM EGCG-treated A549 cells compared to controls were summarized in Table 2. The corresponding results between the 40 μM EGCG-treated group and the control group are shown in Supplementary Tables S2, S3 respectively.

**TABLE 1:** Significantly altered metabolites among the control and the EGCG-treated groups by ANOVA test analysis with Tukey’s post hoc analysis.

	*p*. value	Tukey’s HSD
4-Imidazoleacetic acid	4.970E-07	40 μM-0 μM; 80 μM-0 μM
Glutathione reduced	3.730E-06	40 μM-0 μM; 80 μM-0 μM
Agmatine	1.160E-05	40 μM-0 μM; 80 μM-0 μM
Cytosine	2.710E-05	40 μM-0 μM; 80 μM-0 μM
Aspartate	1.126E-04	40 μM-0 μM; 80 μM-0 μM
2-Deoxycytidine	1.900E-04	40 μM-0 μM; 80 μM-0 μM
2/3-Aminoisobutyric acid/Dimethylglycine	3.167E-04	40 μM-0 μM; 80 μM-0 μM
Proline	3.484E-04	40 μM-0 μM; 80 μM-0 μM
6-Methyl-DL-Tryptophan	4.916E-04	80-0 μM
Serine	0.001	80-0 μM
R5P	0.001	80-0 μM
2-Methylglutaric acid	0.001	40 μM-0 μM; 80 μM-0 μM
Asparagine	0.001	80-0 μM
Acetohydroxamic acid	0.002	80-0 μM

R5P, Ribose-5-phosphate.

**TABLE 2:** The disturbed metabolites with *p* < 0.05 in 80 μM EGCG-treated A549 cells compared to controls.

Metabolite	*p*. value	Fold change
Agmatine	8.640E-06	0.48
4-Imidazoleacetic acid	2.220E-05	18.13
Glutathione reduced	6.860E-05	0.39
Cytosine	1.360E-04	0.50
2/3-Aminoisobutyric acid/Dimethylglycine	2.450E-04	0.73
Aspartate	3.240E-04	1.26
Serine	0.001	1.33
Proline	0.001	1.47
R5P	0.001	1.46
Acetohydroxamic acid	0.001	0.43
2-Deoxycytidine	0.001	0.48
6-Methyl-DL-Tryptophan	0.002	0.73
Asparagine	0.003	1.69
2-Methylglutaric acid	0.004	0.74
alpha-KG	0.014	0.81
Creatine	0.016	0.85
Cytidine	0.019	1.84
Lauric acid	0.020	1.30
Glycine	0.022	1.10
GA3P	0.023	0.69
Acetylcholine	0.023	0.57
Choline	0.026	0.71
Sorbitol	0.026	1.18
IsoCitrate	0.028	0.77
Imidazole	0.030	1.64
Sarcosine	0.030	0.90
Biotin	0.032	0.71
UDP-GlcNAc	0.035	1.22
ADP ribose	0.036	1.34
Adipic acid	0.037	1.44
Pantothenic acid	0.038	0.88
Amino valerate	0.050	1.80

R5P, Ribose-5-phosphate; alpha-KG, alpha-Ketoglutaric acid; GA3P, glyceraldehyde 3-phosphate; UDP-GlcNAc, Uridine diphosphate-N-acetylglucosamine.

#### Analysis of Metabolic Pathways

**FIGURE 6:**
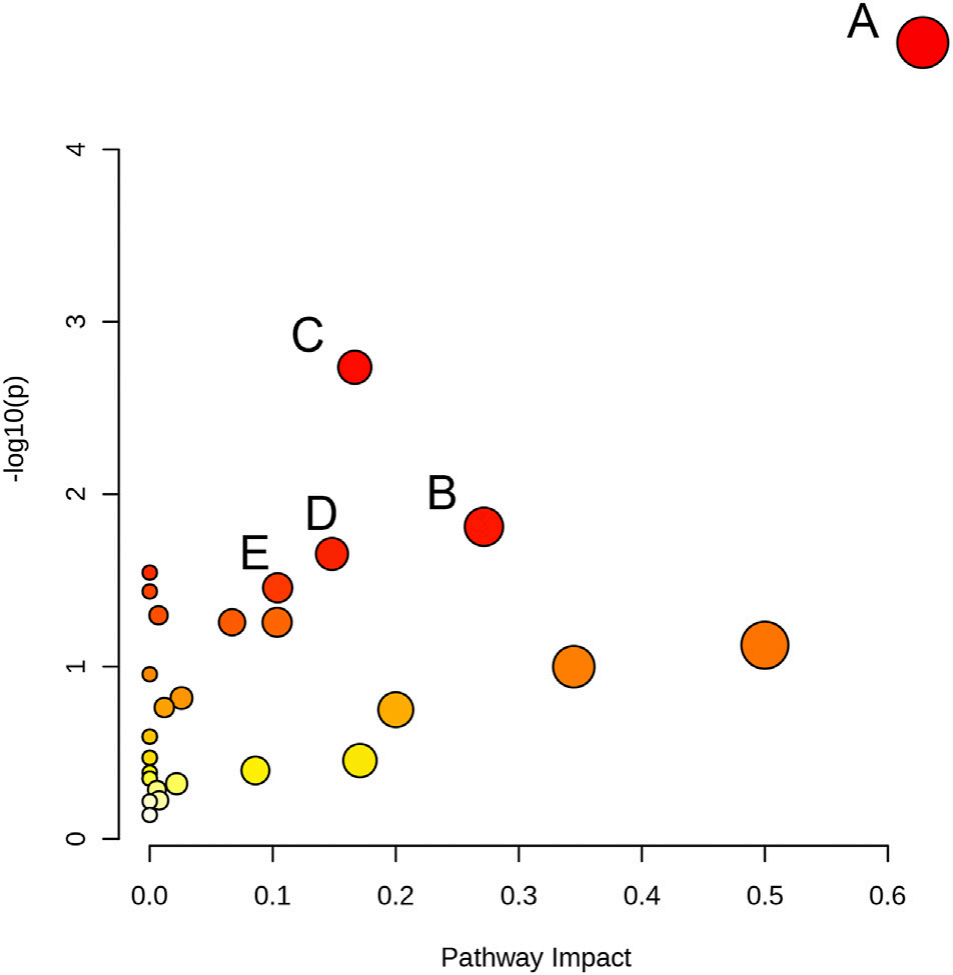
Pathway analysis overview depicting altered metabolic pathways in A549 cells from control and 80 μM EGCG-treated groups. The metabolic pathways are displayed as distinctly colored circles depending on their enrichment analysis scores (vertical axis, shade of red) and topology (pathway impact, horizontal axis, circle diameter) via MetaboAnalyst 5.0. **(A)**: Glycine, serine and threonine metabolism, **(B)**: Alanine, aspartate and glutamate metabolism, **(C)**: Aminoacyl-tRNA biosynthesis, **(D)**: Glyoxylate and dicarboxylate metabolism E: Arginine and proline metabolism.

We used MetaboAnalyst 5.0 and metabolites with *p* < 0.05 to analyze metabolic pathways. Compared to the control group, 27 metabolic pathways were affected (Supplementary Table S4) in the 80 μM EGCG-treated group. Of these, two markedly altered pathways were filtered according to specific criteria (raw *p* < 0.05 and impact value >0.2): Glycine, serine and threonine metabolism (impact value = 0.628), Alanine, aspartate and glutamate metabolism (impact value = 0.272). Each metabolic pathway was represented by a colored circle within the diagram. As shown in Figure 6 and Supplementary Table S4, EGCG induced significant perturbations in Glycine, serine and threonine metabolism, Alanine, aspartate and glutamate metabolism, Aminoacyl-tRNA biosynthesis, Glyoxylate and dicarboxylate metabolism, Arginine and proline metabolism in the 80 μM EGCG group (Figure 6), as well as Histidine metabolism, arginine and proline metabolism in the 40 μM EGCG group (Supplementary Figure S3).

## Discussion

The anticancer ability of EGCG has been shown to be related to its antiproliferative and proapoptotic effects (Ahmad et al., 1997; Khan et al., 2006; Ma et al., 2013; Pal et al., 2018; Almatroodi et al., 2020). Similar to previous reports (Jiang et al., 2016; Li et al., 2016), our data suggest that EGCG concentrations at 40 μM or greater showed evident cell growth inhibition on A549 cells compared to the control group. Considering the balance between cell viability and data interpretability and exploring cell metabolism changes caused by different concentrations of EGCG, 40 and 80 μM were chosen for further experiments in this study, corresponding to 92 and 60% survival rate respectively (Chu et al., 2018; Daskalaki et al., 2018; Zhang et al., 2020). It has been proved that treatment with green tea-based food supplements has acceptable safety, but high doses of EGCG can induce certain toxic side effects (Peter et al., 2017). Intake up to 300 mg EGCG/person/day is a tolerable upper intake level proposed for food supplements (Dekant et al., 2017). In a previous study, the maximum plasma concentration of EGCG was 695.8 ng/ml after receiving oral EGCG in 10 day’s repeated doses of 400 mg (Ullmann et al., 2004).

Studies have demonstrated various biological and pharmacological activities of EGCG, such as antioxidant, anti-inflammatory, antiangiogenic, antiproliferative, proapoptotic, and antimetastatic properties (Gu et al., 2013; Lee et al., 2013; Zhang et al., 2013; Chen et al., 2016; Liu et al., 2016; Fujiki et al., 2017; Almatroodi et al., 2020). *In vitro*, EGCG has been shown to inhibit growth by increasing the percentage of cells at the G0/G1 phase of the cell cycle (Fujiki et al., 2017) and inhibit epithelial-mesenchymal transition and migration via downregulation of HIF-1α, VEGF, pAkt/ERK, COX-2 and vimentin in A549 lung cancer cell (Shi et al., 2015). Also, research has shown that EGCG stimulates apoptosis in the H1299 lung cancer cell line by inhibiting the activation of PI3K/Akt serine/threonine kinase 1 signaling pathway (Gu et al., 2018). One study demonstrated that the inhibition of A549 cell proliferation by EGCG might be achieved via suppressing the expression of the cell death-inhibiting gene, Bcl-xL (Sonoda et al., 2014). Another study showed EGCG also upregulated the expression of the apoptosis-promoting factor Bax by regulating Ku70 acetylation that blocks the interaction between Ku70 and Bax (Li et al., 2016). The amino acids alanine and glutamate were found to be significantly up-regulated in apoptotic HepG2 and HEK293 cells irrespective of the apoptosis inducer (Halama et al., 2013). Disturbed alanine, aspartate and glutamate metabolism in A549 cells under 80 μM EGCG exposure in this study may be related to the proapoptotic effect of EGCG. Long non-coding RNAs (lncRNAs) have emerged as new players in the cancer paradigm. Real-time quantitative reverse transcription-polymerase chain reaction proved a downregulation of *HMMR-AS1, AL392089.1, PSMC3IP,* and *LINC02643* lncRNAs and upregulation of *RP1-74M1.3, AC087273.2, SNAI3-AS1, LINC02532,* and *AC007319.1* lncRNAs in A549 cell lines treated with EGCG (Hu et al., 2019). Various lncRNAs, mRNAs, or proteins regulated by EGCG identified in these studies could affect the metabolic results of A549 cells. Synergistic inhibition of lung cancer cells by EGCG with other drugs has also been reported, such as leptomycin B (Cromie and Gao, 2015), NF-κB inhibitor BAY11-7082 (Zhang et al., 2019), gefitinib (Meng et al., 2019), and cisplatin (Jiang et al., 2016). However, the precise underlying mechanisms of the antitumor activity of EGCG in lung cancer are still largely unclear.

**FIGURE 7:**
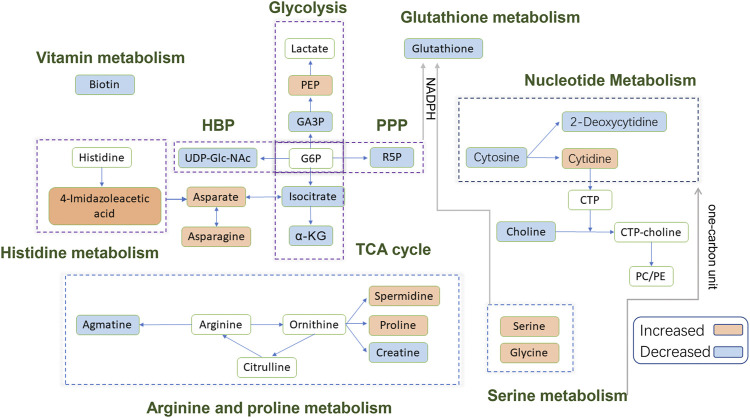
Schematic diagram of the modulated metabolites and potential disturbed metabolic pathways. Up-regulated metabolites detected are shown in the orange background; down-regulated metabolites detected are shown in the blue background; blank background means no statistically significant change or undetected. **Abbreviations:** PEP, phosphoenolpyruvate; GA3P, glyceraldehyde 3-phosphate; G6P, glucose-6-phosphate; α-KG, alpha-Ketoglutaric acid; R5P, Ribose-5-phosphate; CTP, cytidine triphosphate; PC, phosphatidylcholine; PE, phosphatidylethanolamine; HBP, hexosamine biosynthetic pathway; PPP, pentose phosphate pathway; TCA, tricarboxylic acid.

In this study, we used a metabolic approach to further uncover the likely mechanisms underlying the anticancer activity of EGCG in A549 cells. This approach led us to identify 32 differential metabolites (15 upregulated/17 downregulated) in the 80 μM EGCG treated group compared to the control (Table 2). Among the identified metabolites, 11 compounds were significantly changed (fold change >1.5 or <0.75) (Supplementary Table S2). Glycine, serine and threonine metabolism and alanine, aspartate and glutamate metabolism were the two most significantly disturbed pathways under 80 μM EGCG exposure. Histidine metabolism and arginine and proline metabolism were the two most significantly disturbed by exposure to 40 μM EGCG. A schematic diagram of the modulated metabolites and potential disturbed metabolic pathways is shown in Figure 7. A more specific analysis of metabolites is as follows.

### Energy Metabolism

In this study, the metabolomics data suggested that EGCG altered the cellular energy metabolism of A459 cells through glycolysis and the tricarboxylic acid (TCA) cycle. Consumption of glucose by tumors increased markedly compared to the nonproliferating normal tissues to meet the biosynthetic demands associated with proliferation (Warburg et al., 1927). Usually, cancer cells predominantly use glycolysis rather than the TCA cycle for energy production, a phenomenon known as the Warburg effect (Panieri and Santoro, 2016). In previous studies, EGCG significantly reduced lactate production, anaerobic glycolysis, glucose consumption and glycolytic rate in pancreatic adenocarcinoma MIA PaCa-2 cells (Lu et al., 2015). A decrease in glycolysis intermediate glyceraldehyde 3-phosphate was observed in 80 μM EGCG induced cells compared with control cells in this study. However, there were no significant differences in glycolysis intermediates such as glucose-6-phosphate/fructose-6-phosphate (G6P/F6P) and lactate between the control group and EGCG induced group, neither 40 μM nor 80 μM. Interestingly, phosphoenolpyruvate (PEP) was found to increase in the 40 μM group compared to the control. An increase in Glucose-6-phosphate isomerase (GPI), ATP-dependent 6-phosphofructokinase platelet type (PFK-P) and fructose-bisphosphate aldolase A (ALDA) were detected by Wu et al. (2017) in Dox-induced senescent cells compared with control cells, suggesting an up-regulation of the glycolytic pathway during senescence. So, we couldn’t refuse the assumption that the inhibitory effect on glycolysis may be counteracted by induced cell senescence in our research.

Ribose-5-phosphate, which can be generated by the pentose phosphate pathway (PPP), a constituent of nucleotides, was found to increase in the 80 μM group compared to the control. Wu et al. (2017) also found the activation of PPP in senescent cells. We could infer that A549 cells would produce more nucleotide precursors to fulfill the increased need for nucleosides for DNA damage repair by activating PPP when challenged with EGCG treatment.

Our data revealed an elevated level of Uridine diphosphate-N-acetylglucosamine (UDP-Glc-NAc) in the 80 μM EGCG induced group compared to the control one. UDP-Glc-NAc is the end product of a well-established pathway for nutrient sensing-the hexosamine biosynthetic pathway (HBP) and also the donor substrate for modification of nucleocytoplasmic proteins at serine and threonine residues with N-acetylglucosamine (O-GlcNAc) (Wells et al., 2003). Elevated HBP has been reported in cancers and much evidence suggests the HBP helps fuel cancer cell metabolism, growth, survival, and spread (Ferrer et al., 2016; Akella et al., 2019). Interestingly, not only in the cancer cells but also in senescent cells, up-regulation of HBP has been suggested (Wu et al., 2017). The reason for the up-regulation of the UDP-Glc-NAc induced by EGCG in A549 cells needs to be further explored.

The tricarboxylic acid (TCA) cycle is the main pathway of glucose degradation and the primary energy supplier for universal organisms. The 80 μM EGCG induced group showed a down-regulated TCA cycle activity in the A549 cell line, manifested as a decrease in two main TCA cycle intermediates: α-ketoglutarate and isocitrate. However, we didn’t find similar down-regulated TCA cycle intermediates in 40 μM EGCG induced group. This suggested that downregulated TCA cycle in A549 cells induced by EGCG may be dose-dependent and relates to the downregulation of cell viability.

### Amino Acid Metabolism

TCA cycle provides metabolic precursors for the biosynthesis of non-essential amino acids, including aspartate and asparagine. In our study, the level of aspartate and asparagine was increased in 80 μM EGCG induced group, which indicated that there were other ways to supplement the synthesis of aspartic acid.

The significantly increased expression of 4-Imidazoleacetic acid (histidine’s metabolite) was observed in EGCG-treated A549 cells, which implied the disturbance of histidine metabolism. The presented evidence indicates that histamine is an important mediator in cancer development and progression (Rivera et al., 2000), and the effects of histamine’s receptor antagonists on cancer cell proliferation have been explored (Blaya et al., 2010). 4-Imidazoleacetic acid is the most apparent upregulated metabolite among the statistically different metabolites in our study, by 18.13-fold and 5.36-fold in 80 and 40 μM EGCG induced group respectively compared to the control group. 4-Imidazoleacetic acid can be generated from oxidative deamination of histamine and then transform to aspartate. So, the increased level of aspartate and asparagine is not strange in the 80 μM EGCG induced group. Asparagine has also been reported to potentiate CD8^+^ T-cell activation and antitumor responses (Wu et al., 2021). As tumors frequently outgrow their supply, cancer cells reside in oxygen-poor environments. Low oxygen activates a transcriptional program that induces glucose uptake and glycolysis while suppressing the electron transport chain (ETC) activity (Ackerman and Simon, 2014). Studies show that aspartate synthesis plays an essential role in the electron transport chain in cell proliferation (Birsoy et al., 2015; Sullivan et al., 2015). Therefore, aspartate may be a limiting metabolite for tumor growth, and aspartate availability may be targeted for cancer therapy (Garcia-Bermudez et al., 2018).

The metabolites of arginine are involved in multiple pathways. Creatine participates in ATP production, whereas ornithine can be converted to putrescine and spermidine for cell proliferation (Wei et al., 2001; Abraham et al., 2013). Ornithine can also be converted to proline and hydroxyproline for collagen formation and new extracellular matrix deposition (Tan et al., 1983). A meta-analysis of metabolic enzyme expression across diverse tumor types identified pyrroline-5-carboxylate reductase (PYCR1), the principal enzyme in proline biosynthesis, as one of the most commonly overexpressed genes in tumors (Nilsson et al., 2014). Compared with the normal, increased levels of spermidine (in 40 μM EGCG induced group) and proline (both in 40 and 80 μM EGCG induced group) were found in our research. However, the level of CTP itself was not changed. In addition, we found decreased levels of creatine in the 80 μM EGCG induced group, another metabolite of arginine which participates in ATP production (Abraham et al., 2013). Agmatine, which can be converted from arginine by the action of arginine decarboxylase on the cell mitochondrial membrane, was also found to decrease in the EGCG group. Agmatine can induce a decrease in cell proliferation due to decreased intracellular levels of polyamines putrescine, spermidine, and spermine (Higashi et al., 2004). Studies have indicated that agmatine administration to tumor cells *in vitro* results in a suppression of cell proliferation (Molderings et al., 2004; Mayeur et al., 2005). In our research, the antitumor effect of EGCG may counteract the endogenous production of agmatine.

Serine is crucial for multiple metabolic pathways required for cell growth and proliferation, including phospholipid, purine and glutathione biosynthesis, as well as being a methyl source for single carbon metabolism. Serine has been reported to be the third most consumed metabolite by cancer cells after glucose and glutamine (Jain et al., 2012; Dolfi et al., 2013). When a significant amount of serine is converted into glycine, serine releases a one-carbon unit to the one-carbon pool. Glycine could also contribute to the one-carbon pool through the glycine cleavage system. One-carbon pathway metabolites contribute to a number of cellular biosynthetic and regulatory processes. Serine was found to be elevated in our research and glycine slightly, which may indicate the decrease consumption of them for one-carbon unit generation.

### Nucleotide Metabolism

The change of carbon flow in the metabolic stream will cause the abnormality of nucleotide metabolism. The increase of serine level can promote serine-mediated pyruvate kinase 2 (PKM2) activity by inducing allosteric changes of the enzyme (Mazurek, 2011; Chaneton et al., 2012). PKM2 reduces the carbon flux into the serine biosynthesis pathway and the nucleotide biosynthesis pathway, ultimately affecting nucleotide metabolism (Mazurek, 2011; Chaneton et al., 2012). In our study, the decrease of cytosine and 2-deoxycytidine in the 80 μM EGCG-treated group may be related to the up-regulation of serine. This trend is not applicable to cytidine, but it’s not strange when the trend of choline is down-regulated. It has been reported that the reduction of choline and glutathione metabolites is associated with apoptosis (Rainaldi et al., 2008; Halama et al., 2013). Cytidine is a precursor of cytidine triphosphate (CTP) needed in the phosphatidylcholine (PC) and phosphatidylethanolamine (PE) biosynthetic pathways. The down-regulation of choline may lead to a reduction in cytidine consumption.

From Supplementary Table S3, we can find that adenine was downregulated by 0.38-fold which was the most obvious reduction among all the statistically different metabolites in the 40 μM group compare to the control. Clear signaling roles for extracellular adenosine have been established in immunomodulation, vascular remodeling, and promotion of cell growth and proliferation (Chen et al., 2018; Di Virgilio et al., 2018; Morandi et al., 2018; Antonioli et al., 2019). In recent years, it has also been found that adenosine can be used as a signal molecule to affect the biological behavior of tumor cells through different signaling pathways, such as triggering cell cycle arrest, inducing tumor cell apoptosis and affecting cell proliferation (Yang et al., 2007).

### Glutathione Metabolism

In our study, glutathione expression was downregulated in 80 μM EGCG-treated group by 0.39-fold compared with that in the control group. In a metabolomics study of EGCG acting on colorectal cancer cells (HT-29), glutathione expression was also downregulated in EGCG-treated cells (Zhang et al., 2020). Generation of reactive oxygen species (ROS) at high levels can damage nucleotides, proteins and lipids, so impair cell viability. In cancer cells, glutathione oxidation-reduction coupled to NADPH reduction-oxidation is a major pathway for ROS detoxification (Lv et al., 2019). NADPH for ROS turnover through this pathway can be generated from glucose via the pentose phosphate pathway or serine via one-carbon metabolism. As analyzed above, the former one was up-regulated. Taken together, disturbance of glutathione metabolism is a potential pathway involved in the antitumor mechanism of EGCG.

### Vitamin Metabolism

Biotin (vitamin H) is an essential micronutrient vital for normal cellular function (Livaniou et al., 2000). To thrive and multiply rapidly, cancer cells need extra biotin compared with normal cells. Biotin overexpression is observed in wide types of cancer cells, including renal (RENCA, RD0995), leukemia (L1210FR), lung (A549, M109), ovarian (OV 2008; ID8), mastocytoma (P815), and breast (4T1, JC, MMT06056) cancer (Russell-Jones et al., 2004; Chen et al., 2010; Shi et al., 2014). The decreased biotin implied a slowdown in the proliferation of 80 μM EGCG-treated A549 cells compared to the control group.

Finally, there are several limitations in the present study. Firstly, although some previous *in vitro* studies employed the A549 cell line to explore the mechanism of the antitumor effects of EGCG, the A549 cell line cannot represent the true lung cancer cell environment *in vivo*. The concentrations of EGCG from 10 to 100 µM used in most of the studies in cell culture systems, as well as in this paper, are much higher than the concentrations monitored in human plasma (usually lower than 1 µM) after tea ingestion. Thus, it is necessary to verify the high concentration findings in cell lines utilizing lower concentrations in the human body. Secondly, in metabolomics studies, the differences in viability between the control and treated cells would affect the accuracy of the results. The dose of IC50 has been used in the metabolomics research of toxicology in recent years (Yu et al., 2019; He et al., 2020; Hou et al., 2020). To capture more responses on cell metabolism related to the anticancer effect of EGCG and explore the changes in metabolic processes with increasing EGCG concentration, the dosages of 40 and 80 μM were both used in our metabolomics experiment. Thirdly, even though we used a pathway-specific LC-MS/MS method that can cover more than 300 metabolites from over 35 metabolic pathways, there were still many important metabolites left out. This has an impact on the analysis of metabolic pathways. In addition, quantitative proteomics is needed to detect whether there was an increase or decrease in enzymes involved better to explain the upregulation or downregulation of the metabolic pathway. Further experiments are needed to investigate the specific relationship between genetic changes and metabolite changes.

## Conclusion

In this study, the metabolite changes in A549 cells induced by EGCG were investigated utilizing LC-MS-based metabolomics. Our data demonstrated that altered metabolites were involved in the metabolism of glucose, amino acid, nucleotide, glutathione, vitamin and especially associated with serine and threonine metabolism, alanine, aspartate and glutamate metabolism, and histidine metabolism. These findings contribute to understanding the intramolecular metabolic processes of A549 cells caused by EGCG and may provide potential clues for the underlying mechanisms of the anti-cancer property of EGCG. Further researches are required for the therapeutic application of EGCG in cancer management.

## Data Availability

The original contributions presented in the study are included in the article/Supplementary Material, further inquiries can be directed to the corresponding authors.
