# Supplementary material for: LC–MS Based Metabolomics Study of the Effects of EGCG on A549 Cells
Source: Front Pharmacol. 2021 Sep 28;12:732716. doi: 10.3389/fphar.2021.732716 (PMC8505700; doi:10.3389/fphar.2021.732716)
Supplement: Supplementary file 1 [file Presentation1.pdf]

Supplementary Material

1 Supplementary Figures and Tables

1.1 Supplementary Figures

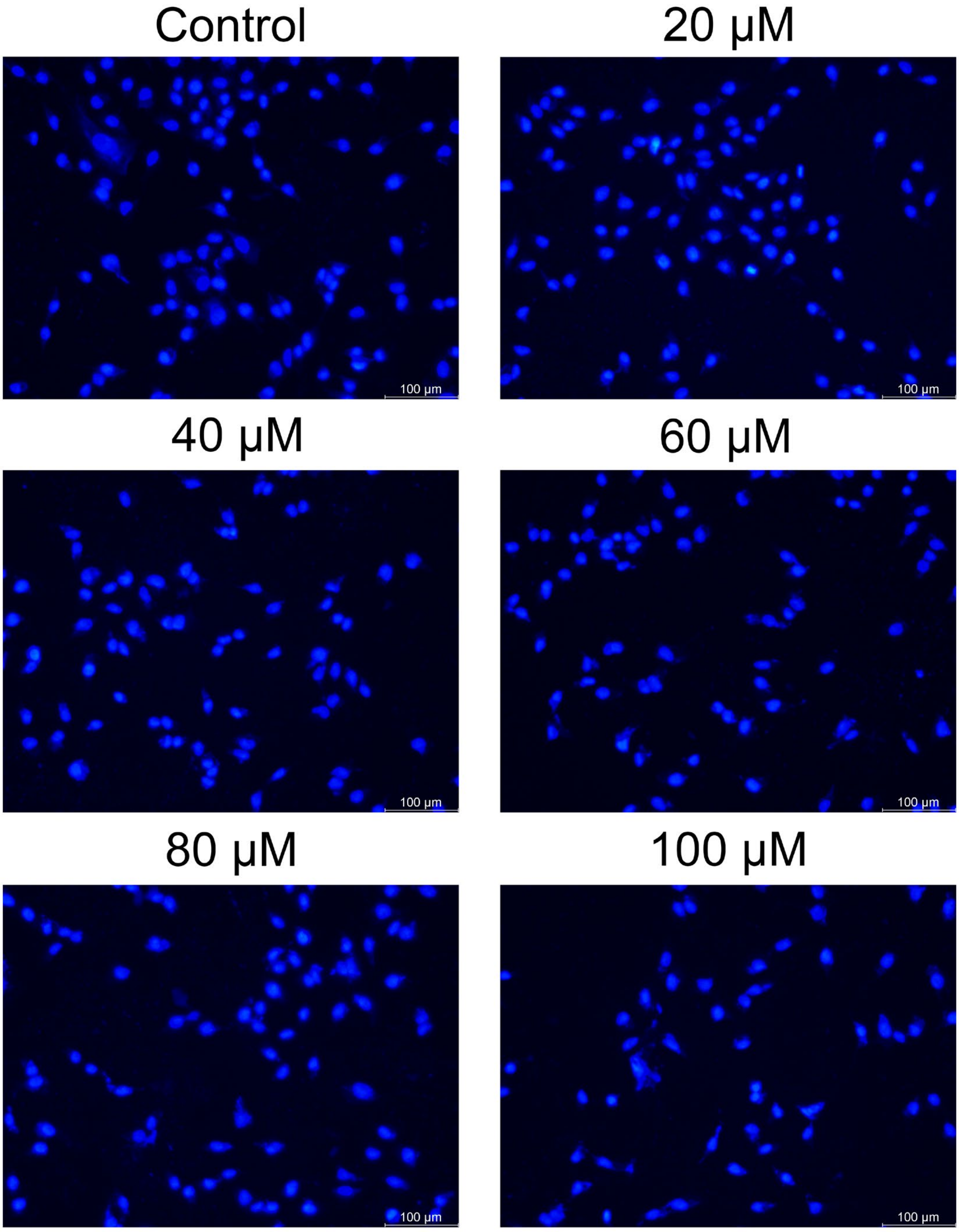

Supplementary Figure 1. Fluorescence staining with 4’, 6-diamidino-2-phenylindole (DAPI) of A549 cells after EGCG treatment for 24h.

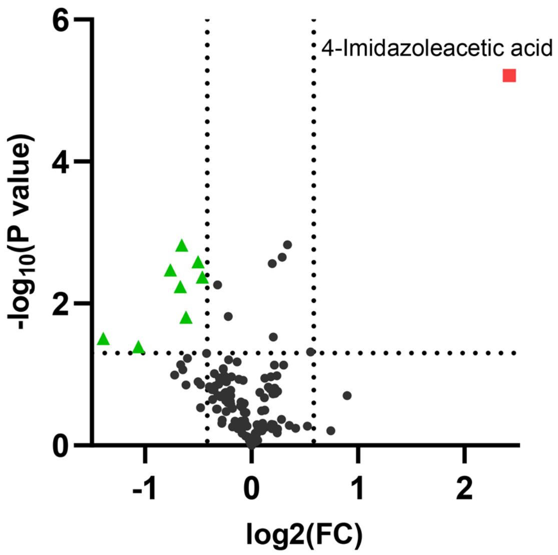

Supplementary Figure 2. Volcano plot analysis of differential metabolites in A549 cells after exposure to 40 μM EGCG. The x-axis represents log2 (fold change), while the y-axis represents p-value in -log 10 scale. The significantly up-regulated metabolites were indicated in red squares, and up-regulated in green triangles. ($p < 0.05$ and fold change > 1.5 or < 0.75).

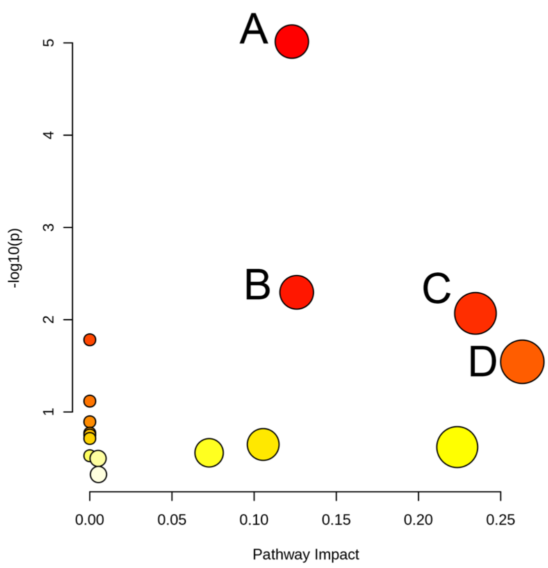

Supplementary Figure 3. Pathway analysis overview depicting altered metabolic pathways in A549 cells from control and 40 μM EGCG-treated groups. The metabolic pathways are displayed as distinctly colored circles depending on their enrichment analyses scores (vertical axis, shade of red) and topology (pathway impact, horizontal axis, circle diameter) via MetaboAnalyst 5.0. A: Histidine metabolism, B: Arginine and proline metabolism, C: Nicotinate and nicotinamide metabolism, D: beta-Alanine metabolism.

1.2 Supplementary Tables

Supplementary Table 1. Potential biomarkers in 80 μM EGCG-treated A549 cells compared to controls.

Metabolite	p.value	Fold Change
Agmatine	8.640E-06	0.48
4-Imidazoleacetic acid	2.220E-05	18.13
Glutathione reduced	6.860E-05	0.39
Cytosine	1.360E-04	0.50
Acetohydroxamic acid	0.001	0.43
2-Deoxycytidine	0.001	0.48
Asparagine	0.003	1.69
Cytidine	0.019	1.84
Acetylcholine	0.023	0.57
Imidazole	0.030	1.64
Amino valerate	0.050	1.80

Supplementary Table 2. Potential biomarkers in 40 μM EGCG-treated A549 cells compared to controls.

	p.value	Fold Change
4-Imidazoleacetic acid	6.150E-06	5.36
Cytosine	0.002	0.64
Agmatine	0.003	0.71
2-Deoxycytidine	0.003	0.59
Glutathione reduced	0.004	0.73
1-Methylhistidine	0.006	0.63
Stearic acid	0.016	0.65
Adenine	0.031	0.38
Urocanic acid	0.040	0.48

Supplementary Table 3. The disturbed metabolites with $p < 0.05$ in 40 μM EGCG-treated A549 cells compared to controls.

	p.value	Fold Change
4-Imidazoleacetic acid	6.150E-06	5.36
Proline	0.001	1.27
Cytosine	0.002	0.64
PEP	0.002	1.22
Agmatine	0.003	0.71
Aspartate	0.003	1.15
2-Deoxycytidine	0.003	0.59
Glutathione reduced	0.004	0.73
2-Methylglutaric acid	0.005	0.80
1-Methylhistidine	0.006	0.63
2/3-Aminoisobutyric acid/Dimethylglycine	0.015	0.86
Stearic acid	0.016	0.65
NAD	0.030	1.15
Adenine	0.031	0.38
Urocanic acid	0.040	0.48
Spermidine	0.048	1.47

PEP phosphoenolpyruvate, NAD nicotinamide adenine dinucleotide

Supplementary Table 4. Metabolite pathway changes with 80 μM EGCG-treated A549 cells compared to controls.

	Total	Expected	Hits	Raw p	Holm p	FDR	Impact
Glycine, serine and threonine metabolism	33	0.63871	6	2.40E-05	0.002014	0.002014	0.62837
Aminoacyl-tRNA biosynthesis	48	0.92903	5	0.001834	0.15224	0.077039	0.16667
Alanine, aspartate and glutamate metabolism	28	0.54194	3	0.01547	1	0.43317	0.27164
Glyoxylate and dicarboxylate metabolism	32	0.61935	3	0.022234	1	0.44081	0.14815
Arginine biosynthesis	14	0.27097	2	0.028532	1	0.44081	0
Arginine and proline metabolism	38	0.73548	3	0.034979	1	0.44081	0.10399
Histidine metabolism	16	0.30968	2	0.036734	1	0.44081	0
Pantothenate and CoA biosynthesis	19	0.36774	2	0.050506	1	0.51756	0.00714
Citrate cycle (TCA cycle)	20	0.3871	2	0.055453	1	0.51756	0.10354
Riboflavin metabolism	4	0.077419	1	0.075271	1	0.63228	0.5
Glutathione metabolism	28	0.54194	2	0.10035	1	0.76633	0.34469
D-Glutamine and D-glutamate metabolism	6	0.11613	1	0.11082	1	0.77576	0
Glycerophospholipid metabolism	36	0.69677	2	0.15228	1	0.98399	0.02582
Biotin metabolism	10	0.19355	1	0.17801	1	1	0.2
Butanoate metabolism	15	0.29032	1	0.25511	1	1	0
Nicotinate and nicotinamide metabolism	15	0.29032	1	0.25511	1	1	0
Fructose and mannose metabolism	20	0.3871	1	0.3252	1	1	0.03313
beta-Alanine metabolism	21	0.40645	1	0.33843	1	1	0
Sphingolipid metabolism	21	0.40645	1	0.33843	1	1	0
Galactose metabolism	27	0.52258	1	0.4127	1	1	0
Porphyrin and chlorophyll metabolism	30	0.58065	1	0.44675	1	1	0
Cysteine and methionine metabolism	33	0.63871	1	0.47889	1	1	0.02184
Amino sugar and nucleotide sugar metabolism	37	0.71613	1	0.51894	1	1	0.00585
Pyrimidine metabolism	39	0.75484	1	0.53784	1	1	0.00505
Primary bile acid biosynthesis	46	0.89032	1	0.59848	1	1	0.00758
Fatty acid biosynthesis	47	0.90968	1	0.60649	1	1	0
Purine metabolism	65	1.2581	1	0.72683	1	1	0
