# Supplementary figures and images for: LC–MS Based Metabolomics Study of the Effects of EGCG on A549 Cells
Source: Front Pharmacol. 2021 Sep 28;12:732716. doi: 10.3389/fphar.2021.732716 (PMC8505700; doi:10.3389/fphar.2021.732716)

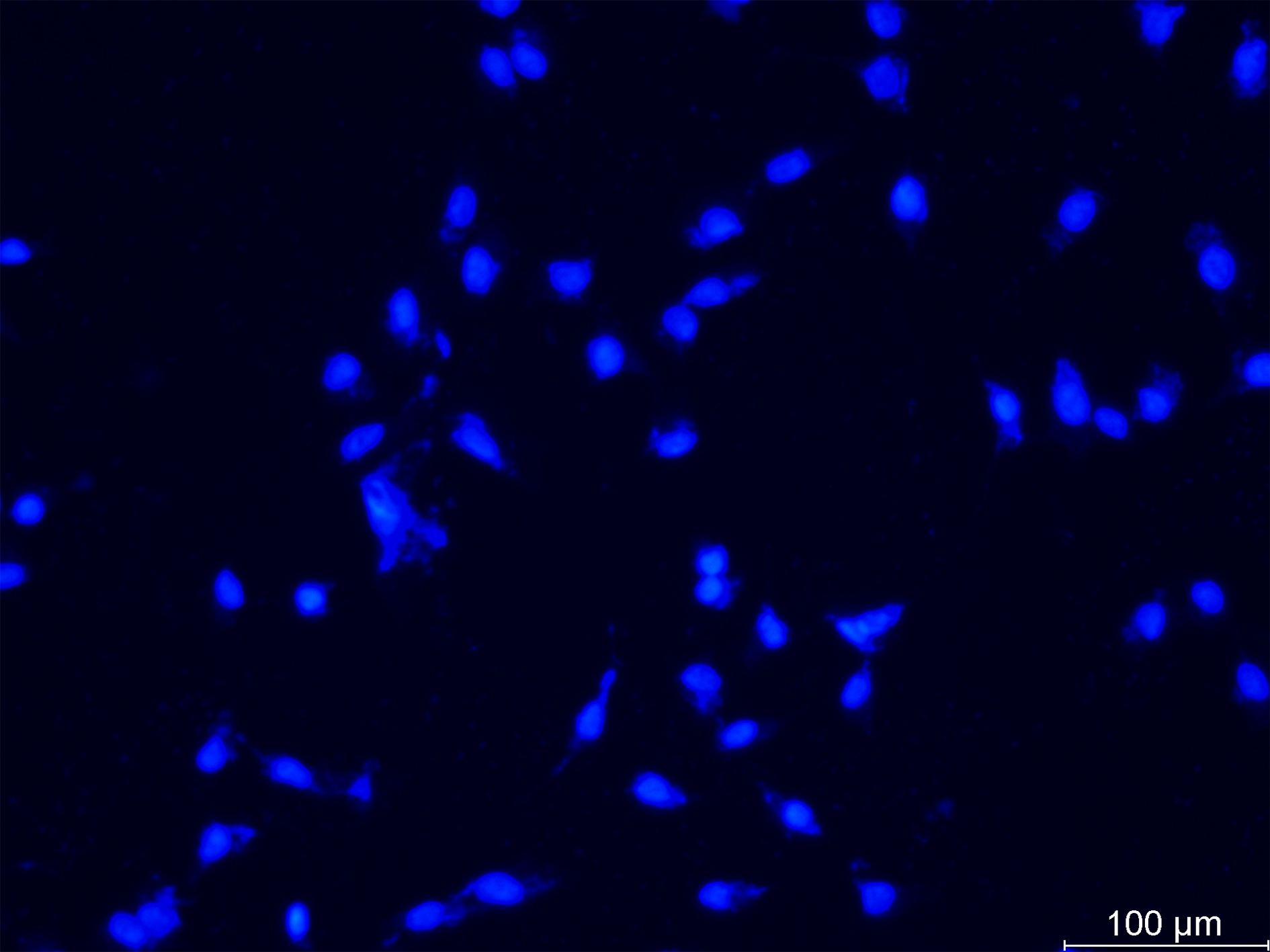

Supplement: Supplementary file 2 [file DataSheet1.ZIP › Original source data/Figures in Supplementary Figure 1/100 μM.tif]

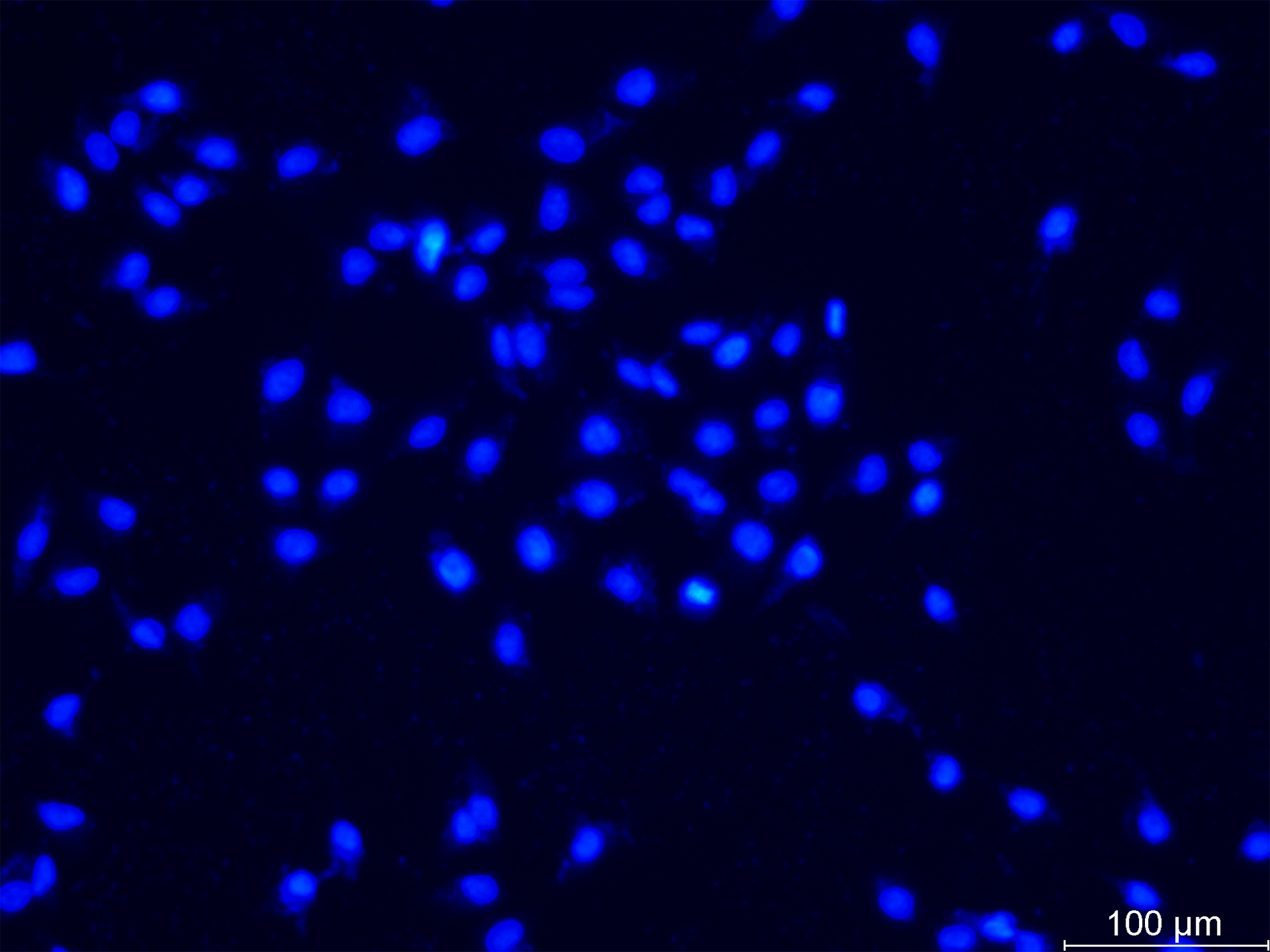

Supplement: Supplementary file 2 [file DataSheet1.ZIP › Original source data/Figures in Supplementary Figure 1/20 μM.tif]

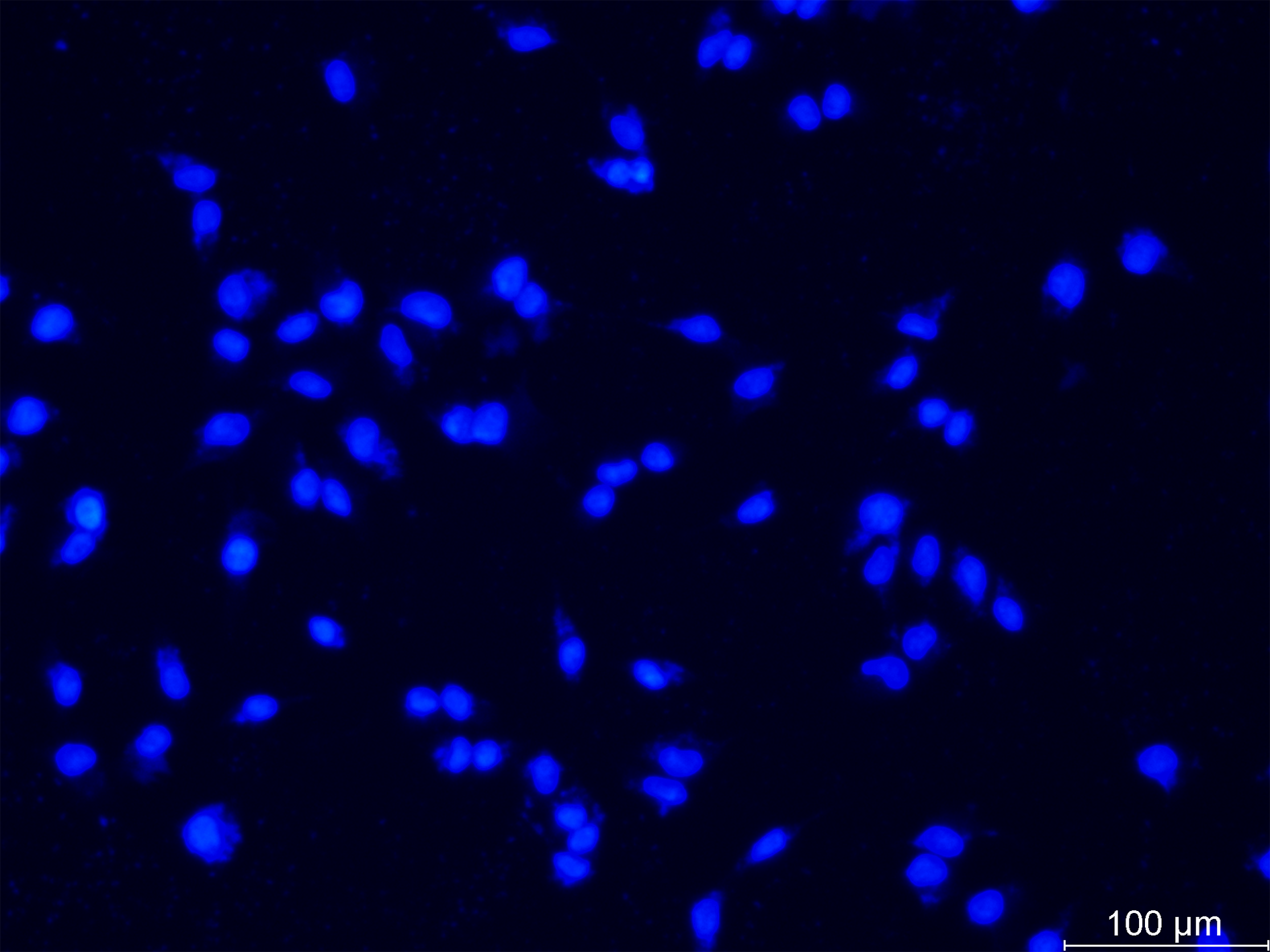

Supplement: Supplementary file 2 [file DataSheet1.ZIP › Original source data/Figures in Supplementary Figure 1/40 μM.tif]

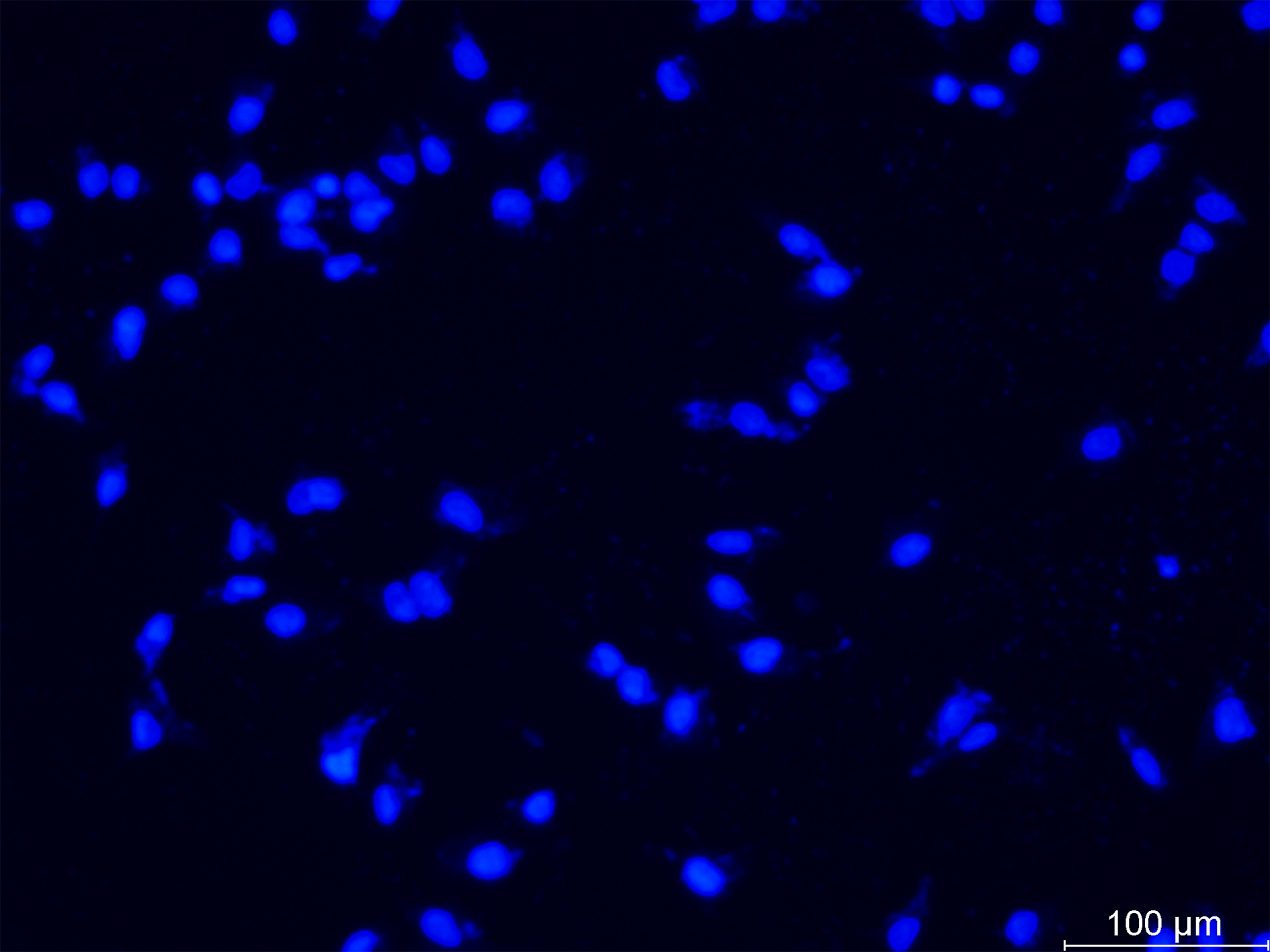

Supplement: Supplementary file 2 [file DataSheet1.ZIP › Original source data/Figures in Supplementary Figure 1/60 μM.tif]

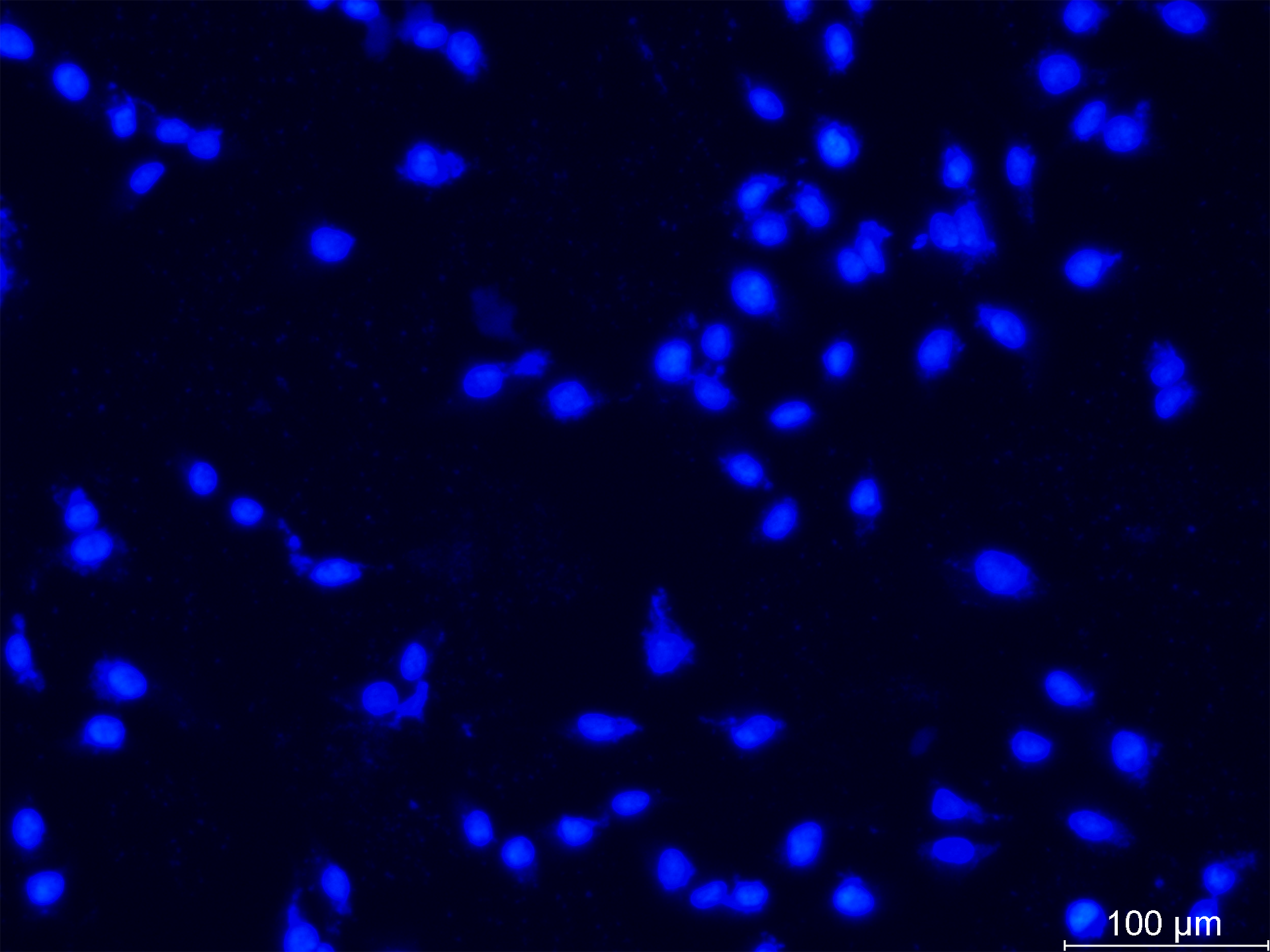

Supplement: Supplementary file 2 [file DataSheet1.ZIP › Original source data/Figures in Supplementary Figure 1/80 μM.tif]

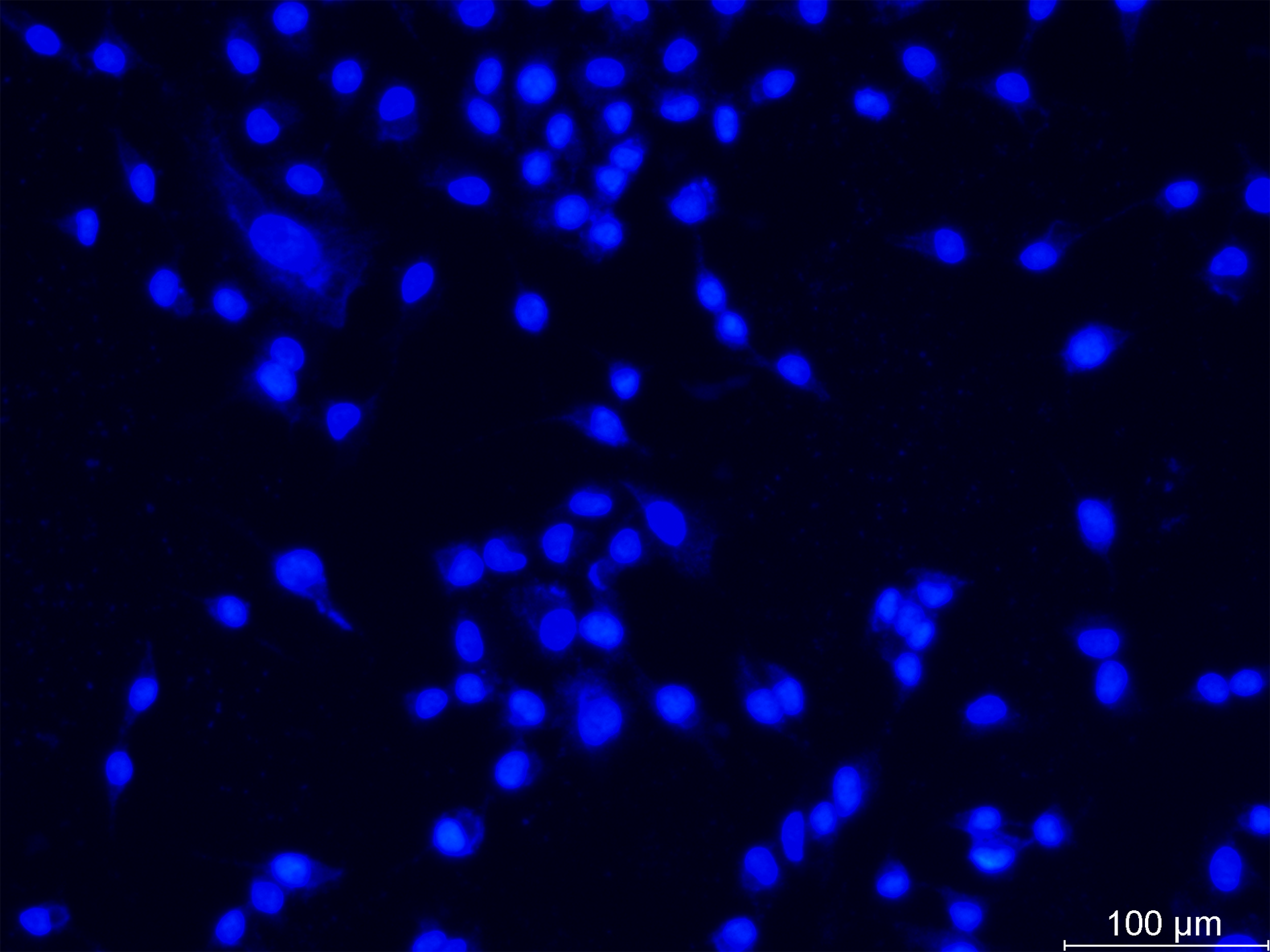

Supplement: Supplementary file 2 [file DataSheet1.ZIP › Original source data/Figures in Supplementary Figure 1/control group.tif]
